# Intraoperative device closure of atrial septal defects in the Older Population

**DOI:** 10.1186/1749-8090-6-123

**Published:** 2011-09-29

**Authors:** Hui Zhang, Qiang Chen, Liang-Wan Chen, Hua Cao, Gui-Can Zhang, Dao-Zhong Chen

**Affiliations:** 1 Department of Cardiovascular Surgery, Union Hospital, Fujian Medical University, Fuzhou, 350001,P. R. China

## Abstract

**Objective:**

This study sought to prove the safety and feasibility of intraoperative device closure of atrial septal defect (ASD) with transthoracic minimal invasion in the older patients.

**Methods:**

From January 2006 to December 2009, 47 patients aged 50 years or more and suffered from atrial septal defect were enrolled in our institution. Patients were divided into two groups, 27 of which in group I with intraoperative device closure and the other 20 in group II with surgical closure. In group I, the method involved a minimal intercostal incision, which was performed after full evaluation of the atrial septal defect by transthoracic echocardiography, and the insertion of the device through the delivery sheath to occlude the atrial septal defect.

**Results:**

In group I, implantation was ultimately successful in all patients. The complete closure rate at 24 hours and 1 year were 81.5% and 100% respectively. In 6 of 27 patients, minor complications occurred: transient arrhythmia (n = 5) and blood transfusion (n = 3). In group II, all patients were closured successfully; almost all of them needed blood transfusion and suffered from various minor complications though. During a follow-up period of 1 to 5 years, no residual shunt, noticeable mitral regurgitation, significant arrhythmias, thrombosis, or device failure were found. In our comparative studies, group II had significantly longer ICU stay and hospital stay than group I (p < 0.05). The cost of group I was less than that of group II(p < 0.05).

**Conclusions:**

Minimally invasive transthoracic device closure of the atrial septal defect at advanced age with a domestically made device without cardiopulmonary bypass is safe and feasible under transthoracic echocardiographic guidance. It was cost-savings, yielding better cosmetic results and leaving fewer traumas than surgical closure. Early and mid-term results are encouraging. However, it is necessary to evaluate the long-term results.

## Background

Atrial septal defect(ASD) is one of the most common congenital cardiac defects and accounts for approximately 6% to 10% of all congenital cardiac defects [1,2]. Most of the patients with ASD are usually asymptomatic and could wait for elective surgical or catheter-based closure for over 3 years. However, due to the poor medical resources and knowledge, some patients with ASD had not been able to receive the best treatment until they reached old age. This phenomenon is particularly prevalent in low-income countries. Although transcatheter closure with Amplatzer septal occluder and surgical repair have been reliably achieved with no mortality and minimal morbidity, [3-6] and the Khan has reported a series of the transcatheter closure in the older patients, [7] the catheter-based closure needs"selective and suitable"patients, more advanced equipments and exposure in X-ray as well. The older patients usually have big size atrial septal defect and the deficiency of the ASD rim, thus closing such patients in the catheterization laboratory could still be quite challenging. The surgical method needs the utilization of CPB and the about 20 cm midline incisions [6]. Meanwhile, since these patients usually suffer from poor health condition and mild-moderate pulmonary hypertension, surgery and CPB for these patients could mean high-risk. With the recent surge of minimally invasive techniques, our approach is to use an intraoperative device and a small incision to close ASD without CPB, which could result in better cosmetic incisions than open-heart surgery. This technique is simple, easy to learn and cost-acceptable in the low-income nations. The aim of this study is to evaluate the safety and feasibility of intraoperative device closure of ASD in the older patients with transthoracic minimal invasion. The results are encouraging [8,9].

## Materials and methods

The present study was approved by the ethics committee of our university and adhered to the tenets of the Declaration of Helsinki. Additionally, the written informed consent was obtained from the patients.

### Device

**Figure 1:**
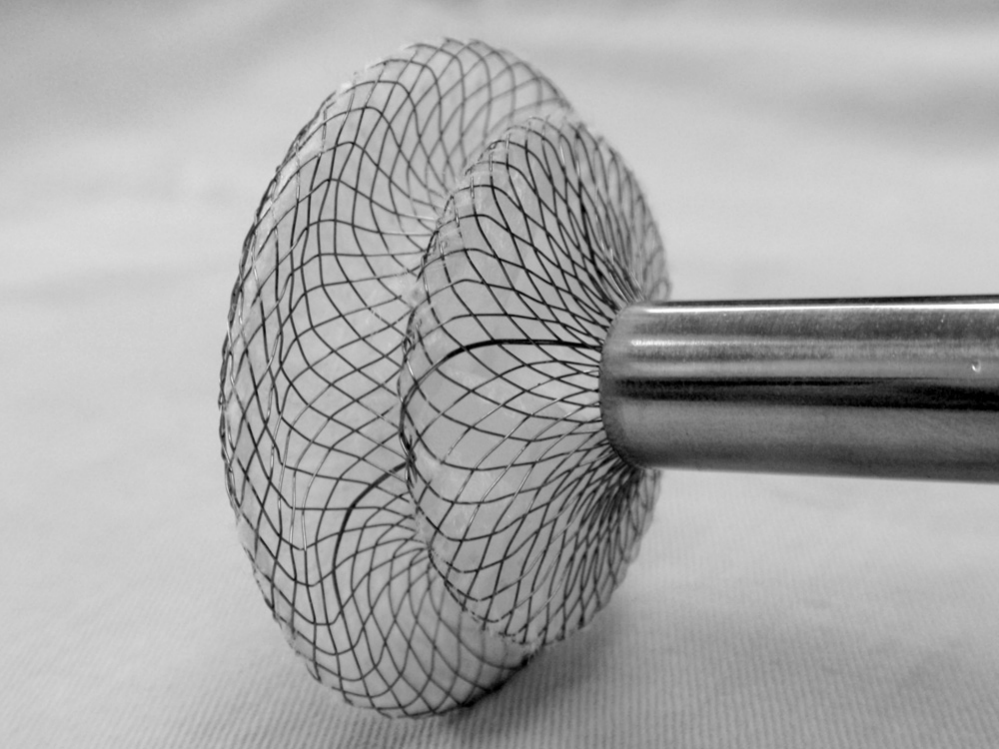
**The occlusion devices**.

The ASD occluder was modified from the Amplatzer atrial septal occluder. It was made in Dong Guan Ke Wei Medical Apparatus Co.Ltd of China. [Figure 1] The device consists of an occluder made from an alloy of nickel and titanium, a metal sheath, a pushing rod, and a hook. The double disc occluder has a loop on the right disc with a 100-cm thread through the loop, facilitating its withdrawal into the 40-cm long and 8-10 mm diameter sheath. The occluder was selected in accordance with the corresponding transthoracic echocardiography result, a maximum defect diameter plus 2-6 mm. The occluder was loaded into the sheath [8-10].

### Patients

The patients were divided into two groups in accordance with their own choices of the methods of closure. No difference in age and body weight distribution was found in either group. The participants were enrolled in our institution between January 2006 and December 2009. Those with other coexisting cardiac anomalies were excluded from our study. Transthoracic echocardiography was used to confirm the diagnosis of the atrial septal defect in those patients and assess the circumferential margins. Routine examinations included a standard electrocardiogram, a chest X-ray, and blood tests. All patients were symptomatic, which included frequent respiratory infection, palpitations, shortness of breath, exercise intolerance and insignificant chest pain. Indications for the atrial septal defect closure were the same as those used for surgical closure, which included hemodynamically significant left to right shunts and (or) significant chamber enlargement, and (or) mild to moderate to severe pulmonary hypertension, despite medical therapy and (or) history of infective endocarditis.

Group I included 27 patients (15 males and 12 females) with isolated ASD received intraoperative device closure treatment without CPB. The patients were aged from 50 years to 65 years (mean ± standard deviation, 56.5 ± 4.2 years). Their weights ranged from 48 to 75 kg (61.6 ± 7.1 kg). Of all patients, 12 patients had mild pulmonary hypertension (which was assessed by TTE, pulmonary artery systolic pressure 30-45 mmHg). 13 patients had moderate pulmonary hypertension (pulmonary artery systolic pressure 45-75 mmHg), and 2 patients had severe pulmonary hypertension (pulmonary artery systolic pressure 75 to 90 mm Hg). 10 patients were diagnosed as ASD with the inferior vena cava rim deficiency.

Group II included 20 patients (12 males and 8 females) who refused device closure and received surgical closure. Their ages ranged from 50 years to 64 years (56.6 ± 4.4 years) and their weights ranged from 50 to 72 kg (60.3 ± 5.7 kg). Of all patients, 10 patients had mild pulmonary hypertension. 9 patients had moderate pulmonary hypertension, and 1 patient had severe pulmonary hypertension.

### Protocol

**Figure 2:**
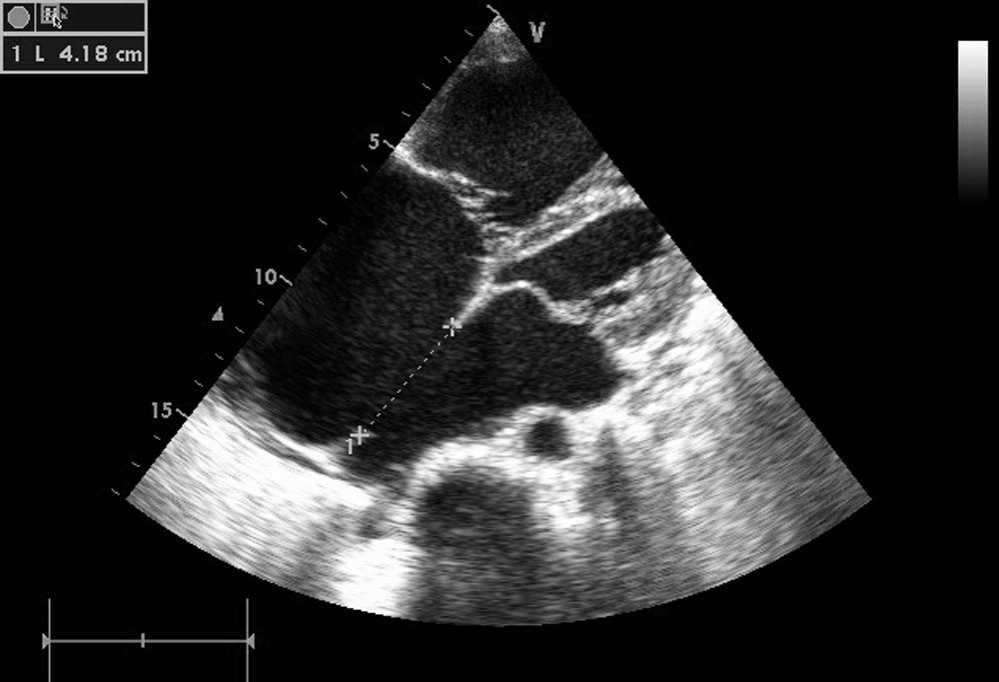
**The 42 mm ASD measured by TTE**.

**Figure 3:**
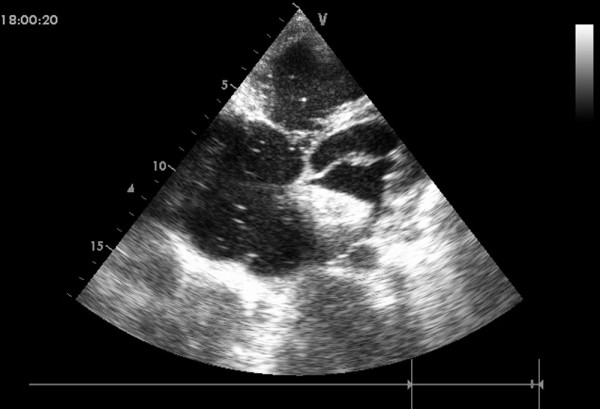
**The sheath positioned from the right atrial free wall into the left atrial cavity across the ASD**.

**Figure 4:**
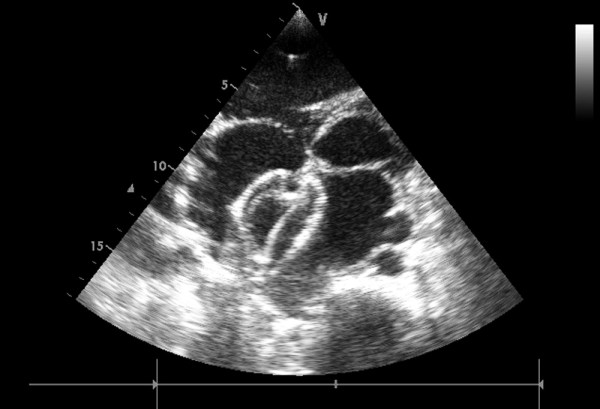
**Final image shown after the 44 mm occluder were deployed and the sheath was withdrawn**.

In group I, general anesthesia was applied to patients and then they were placed in a supine position and draped for exposure of the entire chest with the right hemithorax elevated to approximately 30 degrees. Intraoperative transthoracic echocardiography (TTE) was used to assess the ASD, especially the defect size and circumferential margins adjacent to the superior vena cava, inferior vena cava, pulmonary vein, mitral valve, and aortic sinus [11]. The atrial septal occluder was chosen according to the largest diameter of the ASD, [Figure 2] allowing for a margin of 2-6 mm in excess of the diameter in patients. A right anterior submammary minithoracotomy (about 5 cm in length) was made through the fourth intercostal space. A small rib spreader was used in this manipulation incision to facilitate instrumentation. The pericardium was opened and suspended to expose right atrium. In the anterolateral right atrium, two parallel 4/0 Prolene sutures of approximately 15 mm in diameter were stitched. Heparin was intravenously given at 1 mg/kg body weight, and the activated clotting time was monitored to be greater than 250 sec. The occluder was drawn into the delivery sheath, then a 15 mm incision was opened in the right atrium and the delivery sheath was inserted. Under continuous TTE guidance, the sheath was advanced through the ASD into the left atrium. [Figure 3] The left disc was deployed first by pushing the rod. Adjusting the left disc to be parallel to the atrial septum, the sheath was withdrawn, and then the right disc was deployed on the other side to occlude the ASD. [Figure 4] A to-and-fro motion of the sheath was performed to ensure a secure position across the defect [12,13]. In those patients with the inferior vena cava rim, the occluder would been easily dislodged back into the right atrium thought the deficienct inferior vena cava rim, some new technique were therefore added [9]. During the process of the occluder deployment, the occluder was moved to the rim of superior vena cava by moving the sheald as close as possible, then the "left atrium-occluder-the right atrium" suture through the junction of the Waterston's groove and the inferior vena cava was made in order to fix occluder. After the TTE evaluated there were no significantly residual shunt, no atrioventricular valve distortion and no obstruction of the coronary sinus, the thread was cut and the sheath was withdrawn with the suture snugly tied. The chest was closed routinely with a drainage tube placement. Oral aspirin had been taken for 3 months as an anticoagulation.

In group II, all patients had been attempted open-heart repair with a median sternotomy about 20 cm incision and cardiopulmonary bypass.

### Statistical Analysis

Continuous data are given as mean ± standard deviation and range. Clinical parameters between the two groups were compared with the independent samples t-test. A p value of < 0.05 was defined as statistical significance.

## Results

**Figure 5:**
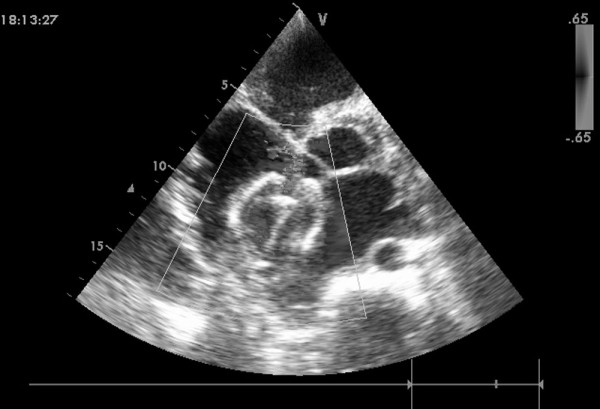
**Trivial or small residual shunts in the edge of the occluder which can be ignored**.

In group I, delivery of occluder was successful in all patients. The size of the ASD as measured by transthoracic echocardiography (TTE) ranged from 32 to 42 mm (mean 36.5 ± 2.7 mm). The size of the occluder implanted ranged from 34 to 46 mm, (mean 34.6 ± 4.8 mm) and the diameter of the sheath was 8-10 mm. The duration of the procedure was in 40-70 minutes (mean 55.8 ± 8.6 minutes). The intensive care unit stay was about 10-24 hours(mean 14.2 ± 3.7 hours), and hospital stay was 6-12 days (mean 7.7 ± 1.5 days). In those who had a successful attempt, the overall immediate complete closure rate was 81.5%. A tiny smoke-like residual flowing through the device or the junction of the occluder and the rim of the ASD were seen immediately after the procedure was implemented in 5 patients. [Figure 5] At 3 months, 1 out of 27 patients still have small residual shunts. However, the closure rate remained 100% at 1 year's follow-up.

Minor complications were encountered in some patients, including transient arrhythmia(n = 5) in the course of the device deployment. Temporary sinus bradycardia, atrial premature beats and transient left bundle branch block were observed in these patients immediately, which were easily treated by drugs or spontaneous recovery. Immediate postprocedure third-degree AVB was observed in 1 patient with an ASD diameter of 40 mm and an occluder size of 44 mm. Since heart rates were ranging about 50-55 bpm, no intervention except closed observation was needed. After treated by glucocorticoid, the AVB resolved spontaneously after 1 week. 2 patients with post-operative cardiac arrest were successfully cardiopulmonary resuscitated. These 2 patients had preoperative left bundle branch block and atrioventricular block. After occlusion, transient left ventricular volume overloaded and slow heart rate may result in cardiac arrest. After treatment of dopamine and furosemide for several days, both patients recovered well. 5 patients need to be retrieved the device several times, which occurred in the apparent residual fistula and the deficiency of the rim. In 3 cases, blood loss requiring transfusion occurred. Therefore, it is important to prepare plenty of blood products before the operation starts. Large pleural effusion occurred in 3 patients and they were treated with drainage tube placement. 3 patients developed mild tricuspid regurgitation. There had been no episodes of endocarditis, thromboembolism, device disruption or failure, mitral valves distortion, complete atrioventricular block or permanent rhythm disturbances.

In those who had a successful attempt, total follow-up period ranged from 1 to 5 years (2.5 ± 1.1 years). Out-patient follow-up was conducted by functional, echocardiographic, and ECG assessment. Symptoms had been either resolved totally or improved significantly in all symptomatic patients. Those patients with mild-moderate pulmonary hypertension had significantly decreased as evaluated by the tricuspid regurgitation jet. 2 patients were still in moderate-severe pulmonary hypertension after operation and needed long-term drug treatment such as inhale Iloprost and/or oral Sildenafil. Symptoms such as palpitations, shortness of breath and exercise intolerance had been improved significantly in these 2 patients. However, long-term effect needs further follow-up. The overall immediate complete closure rate was 81.5%. Those patients have small residual shunts and the position of the shunt was the junction of the occluder and the rim of defects or the device itself. However, the closure rate remained 100% at 1 years' follow-up. No progressive moderate -severe pulmonary regurgitation occurred. Neither thromboembolic event nor other major complications were found during follow-up period. To date, none of the patients in our group has developed complete heart block or/and mitral regurgitation. The incision in the chest was minor and cosmetic.

In group II, all patients needed blood transfusion. Arrhythmia and atrial premature beats were observed in 15 patients, especially during the operation. Some patients would recover immediately while others needed medicine treatment for 3-5 days. 2 patients had Mobitz type II or complete atrioventricular block and were treated with temporary epicardial pacemaking. All of them eventually resumed sinus rhythm after treated by glucocorticoid. 3 patients with large pleural effusion needed place drainage tube. 2 patients suffered from postoperative serious pulmonary infection and cardiac dysfunction. During follow-up period (2.4 ± 1.1 years), there were no episodes of ASD residual fistula, hydrothorax, endocarditis, thromboembolism, or permanent rhythm disturbances. The incision in the median chest was about 20 cm.

Table 1 demonstrates the clinical data comparison of all patients in both groups. Group II required longer operative time, ICU stay and hospital stay than group I (p < 0.05). The average total cost in the surgical group (24084 ± 1219.8 RMB) was higher compared with that in the device group (20289.9 ± 884.8 RMB) (p < 0.05).

**Table 1:** Comparison of clinical data in both groups.

	Group I	Group II	p value
**Number of patients**	27	20	
**Male/female**	15/12	12/8	
**Age (years)**	56.5 ± 4.2	56.6 ± 4.4	P > 0.05
**Body weight(kg)**	61.6 ± 7.1	60.3 ± 5.7	P > 0.05
**ASD size(mm)**	36.5 ± 2.7	35.9 ± 3.6	P > 0.05
**operative time(minutes)**	55.8 ± 8.6	99 ± 9.4	P < 0.05
**ICU stay (hours)**	14.2 ± 3.7	15 ± 4.5	P < 0.05
**Hospital stay (days)**	7.7 ± 1.5	9 ± 1.9	P < 0.05
**Follow-up(years)**	2.5 ± 1.1	2.4 ± 1.1	P > 0.05
**Total cost (RMB)**	20289.9 ± 884.8	24084 ± 1219.8	P < 0.05

## Discussion

Patients with secundum ASD are usually asymptomatic. However, due to the poor medical resources and knowledge, some patients with ASD have not received best treatment until they are old. This phenomenon is prevalent in low-income countries. Elective open-heart repair with midline sternotomy and cardiopulmonary bypass has been considered as the golden standard for the closure of the ASD. Although surgical closure has been proved safe and effective, it is still associated with midline sternotomy and cardiopulmonary bypass in a longer hospital stay. The midline incisions would also reserve the physical and psychological trauma for the patients in their future. Meanwhile, there have been only a few reports about surgical repair of the ASD in the older patients until now. In addition, old patients are usually complicated by pulmonary arterial hypertension and bad body condition, which means more risk to surgery and CPB. With the development of various devices, percutaneous transcatheter occlusions of ASD gradually become another choice for selected patients, especially those with secundum ASD. Clinical experience with device closure of ASD is increasing, and complications seem to be limited in various studies. Compared with those of surgical approaches, device closure has the advantage of bringing less discomfort to patients and leaving no incisional scar. However, due to the big size or the deficient rim of the ASD of those older patients, percutaneous approach sometimes could not be utilized for closure. A certain failure rate percentage exists in those patients with big size or the deficient rims of ASD, such as residual shunts, subsequent malposition and embolization of the device. Furthermore, due to expensive equipments and high cost might be required, many hospital in the low-income nations have no resources on developing this technology. Thus, we applied a minimally invasive technique, intraoperative device closure of those older ASD patients, imitating percutaneous closure of ASD.

Closure of ASD is often considered non-beneficial in older patients because of the lack of experience. According to Khan's report, [7] transcatheter device closure of ASD in adults over the age of 40 years is not only safe and effective, but also results in symptomatic relief by improving functional class and 6MWT distance with favorable cardiac remodeling. Our approach may have a role in a subset of the older patients who have defects which may be difficult to close percutaneously but have high risk for surgery closure with CPB. We applied the same criteria with cardiologists to allow us to choose the "suitable" ASD for the device closure in those older patients. Our recommendations are to use the device closure in those patient with hemodynamically significant L-R shunts and/or significant chamber enlargement, and/or mile-moderate pulmonary hypertension. The open-chest approach offered an operative field and allowed the cardiac surgeons to do better with traditional surgical techniques. Because of the avoidance of CPB, we could limit the length of incision to about 5 cm. Moreover, it was easy to extend for conversion to a regular open-heart procedure once the intraoperative device closure failure without additional incisions or rearranging the operation from the catheterization laboratory to the operating room, which ensures the safety of the procedure. The procedure time could be significantly shortened; the skin-to-skin time could be limited to 40-70 min. Our method provided a perpendicular angle to the atrial septum, which may result in more easily deploying the occluder into the defect than the percutaneous device closure method. A dilemma for an ASD is choosing the location for the placement of the occluding device, especially for the older patients with big size or the deficient rims of ASD. Since larger device may interfere with the neighboring structure, the device size and position must be verified carefully by using TTE before and after deployment. The device selected should be 2-6 mm larger than the ASD, as measured by TTE. When our methods are adopted, it is advisable to retract the right disc into its sheath and push the sheath by hand and move the occluder to a suitable position, and then reopen the right disk. This process needs to be repeated for many times until success during the operation. Based on our experience,[8] the occluder would been dislodged back into the right atrium, so it's necessary to observe the location of the occluder for about 10-20 minutes after the occluder was released. Sometimes, during the process of the occluder deployment, the occluder was moved to the sufficient rims by moving the sheath as close as possible, then the "left atrium-occluder-the right atrium" suture through the Waterston's groove was made in order to fix the occluder. Although some patients had small residual shunts and the position of the shunt was the junction of the occluder and the deficient rim, endothelialization would cover the surface of the device and neointima would form and would fully close any residual shunting after several weeks.

Transthoracic echocardiography is the primary tool for detecting ASD. TTE with Doppler study can be used for measurement of flow dynamics, assessment of the direction and magnitude of abnormal flow across an ASD, and acquisition of the Qp/Qs ratio. Accurate measurement of the size of the defect and surrounding rims by TTE was essential for intraoperative device closure. Although as Sheung-Fat Ko has reported that compared with the findings at cardiac CT and TEE, there are no significant differences in the three techniques in assessment of the long axis of small ASDs, underestimation of the long-axis of large ASD was common at TTE [14]. In our opinion, TTE may afford a good visualization of ASD and its spatial relations with neighboring structures. With the experienced operators, TTE is also a reliable method in quantitating ASD diameters and guiding device-deployment procedures. Usually, we choose large size device, so that we don't consider the underestimation of the long-axis of large ASD [8,9].

Atrial septal defects with a left-to-right shunt lead to development or deterioration of volume overload right ventricular hypertrophy and failure or pulmonary hypertension. Such factors might explain the unfavorable course in the older patients' ASD closure [15]. Those ASD closure might lead to pulmonary congestion or even edema in the older patients, as well as to aggravation of right heart failure in those patients with pulmonary hypertension. Bruch's report on a series of 15 patients with advanced age and/or left or right heart failure and/or pulmonary arterial hypertension using fenestrated ASD occlusion shows that high-risk ASD occlusion can be safely accomplished with excellent clinical results and without complications by a fenestrated occluder [16]. In our study, those older patients with moderate-severe pulmonary hypertension inhale Iloprost and/or oral Sildenafil for 3-6 months before ASD closure [17]. After drug treatment, our study group had shown significant improvement in 6MWT distance, which is useful in the serial evaluation of patient status. The most clinically relevant finding was the increase in exercise capacity as indicated by the increase in 6MWT distance. Qp/Qs was reduced significantly, indicating the reduction in the left-to-right shunting through the ASD, showing a trend toward a reduction in the pulmonary, right atrial, and right ventricular pressures. Then those patients receive device closure and inhale Iloprost and/or oral Sildenafil for 6 months after ASD closure. Symptoms had been improved significantly. No progressive severe pulmonary arterial hypertension and right heart function failure were detected and long-term effect needs further follow-up.

In our series of study, device closure was successful in all patients. In those who had a successful attempt, closure rate was 81.5% immediately after operation, 100% at 12 months follow-up. Trivial or small residual shunts can be ignored immediately after the release of device since they usually disappeared during follow-up period. This early shunting was associated with the loose-links between the occluding device and the rim of defects or the device itself. Several weeks later, endothelialization would cover the surface of the device. Neointima would form and would fully close any residual shunting. Minor complications were encountered in some patients, including transient arrhythmias(n = 5) in the course of the occluder deployment. Temporary sinus bradycardia and atrial premature beats were observed in these patients immediately, which were easily treated by medicine or automatic recovery [18]. The atrial septal would possibly be deformed when the occulder released, which may temporarily affect the heart conduction system. The occluder we chose must be large enough to close the ASD, but not to change cardiac geometry structure. When the occlude gets larger, the atrial septal would be more easily deformed. If any deformation or interference of the device were found, redeployment of the device would have to be undertaken. Reducing intraoperative stimulus was also beneficial for transient arrhythmias. In studies involving transcatheter closure, comparison of successful and unsuccessful deployment revealed a significant association between the deficiency of the rim and a large defect diameter with failure of implantation [19,20]. In our study, we did not encounter failure in the implantation of the device using intraoperative device closure of atrial septal defects.

Relatively higher medical cost always poses a real challenge in popularizing a percutaneous approach in the low-income nations. A domestically made device was chosen so as to maximally reduce the medical costs. This technique did not need expensive X-ray machine, and could also be easily mastered and minimized the operative time. However, it is noted that our study was conducted in low-income countries where health care resources were limited. This was a nonrandomized single-center cohort study and was bias associated with data collection and the incomplete data for some patients. As a result of the above mentioned 27 successful closure cases, our experience was limited. Given the limited number of the older patients, a randomized trial is difficult to pursue. Larger scales of research are required and longer follow-up are needed to determine the future research. This study was limited to one institution, and other institutions may find different results.

The study serves to demonstrate that intraoperative device closure is technically feasible and can be performed at low risk in the older patients. It had the advantages of cosmetic results, leaving less trauma than surgical closure. It therefore should be recommended.

## Conflict of interests

The authors declare that they have no competing interests.

## Authors' contributions

HZ collected the clinical data and performed the statistical analysis, participated in the operation and drafted the manuscript. QC participated in the operation and drafted the manuscript, revised and submitted the paper. LWC and HC designed the study and performed the operation. DZC, participated in the operation. GCZ was the technician of echocardiography for intraoperation TEE surveillance and provided the ultrasound images. All authors read and approved the final manuscript.
